# A second riboswitch class for the enzyme cofactor NAD^+^

**DOI:** 10.1261/rna.077891.120

**Published:** 2021-01

**Authors:** Shanker S.S. Panchapakesan, Lukas Corey, Sarah N. Malkowski, Gadareth Higgs, Ronald R. Breaker

**Affiliations:** 1 Department of Molecular, Cellular and Developmental Biology, Yale University, New Haven, Connecticut 06520-8103, USA; 2 Department of Chemistry, Yale University, New Haven, Connecticut 06520-8103, USA; 3 Department of Molecular Biophysics and Biochemistry, Yale University, New Haven, Connecticut 06520-8103, USA; 4 Howard Hughes Medical Institute, Yale University, New Haven, Connecticut 06520-8103, USA

**Keywords:** aptamer, gene control, *pnuC*, nicotinamide adenine dinucleotide, nicotinamide riboside transporter, noncoding RNA

## Abstract

A bacterial noncoding RNA motif almost exclusively associated with *pnuC* genes was uncovered using comparative sequence analysis. Some PnuC proteins are known to transport nicotinamide riboside (NR), which is a component of the ubiquitous and abundant enzyme cofactor nicotinamide adenine dinucleotide (NAD^+^). Thus, we speculated that the newly found “*pnuC* motif” RNAs might function as aptamers for a novel class of NAD^+^-sensing riboswitches. RNA constructs that encompass the conserved nucleotides and secondary structure features that define the motif indeed selectively bind NAD^+^, nicotinamide mononucleotide (NMN), and NR. Mutations that disrupt strictly conserved nucleotides of the aptamer also disrupt ligand binding. These bioinformatic and biochemical findings indicate that *pnuC* motif RNAs are likely members of a second riboswitch class that regulates gene expression in response to NAD^+^ binding.

## INTRODUCTION

Most known riboswitch classes sense compounds that are derived from RNA monomers or their precursors (McCown et al. 2017). This finding is consistent with the hypothesis that some of these metabolite-sensing noncoding RNAs might have an ancient origin, and sense compounds that were likely relevant during the RNA World (Benner et al. 1989). For example, nucleotide-like enzyme cofactors are ligand targets for the largest collection of riboswitch aptamers, and there are at least 16 classes known to date that sense these coenzymes (McCown et al. 2017; Mirihana Arachchilage et al. 2018; Atilho et al. 2019; Chen et al. 2019; Malkowski et al. 2019).

A recent report (Malkowski et al. 2019) presented bioinformatic, genetic, and biochemical data supporting the hypothesis that *nadA* motif RNAs (Weinberg et al. 2017) function as riboswitches for the coenzyme NAD^+^. This discovery helped overcome the strange circumstance that NAD^+^, one of the most abundant, ubiquitous, and ancient coenzymes in biology, had no identified corresponding riboswitch classes (Breaker 2011; Malkowski et al. 2019). In contrast, nearly all other coenzymes are sensed by one or more distinct riboswitch classes that regulate genes related to the transport, synthesis, or use of these molecules (McCown et al. 2017).

The known NAD^+^-sensing riboswitches based on the *nadA* motif are rare (101 representatives) and limited to the phylum Acidobacteria (Malkowski et al. 2019), whereas many thousands of representatives are known for some riboswitch classes that sense other coenzymes (McCown et al. 2017). The relative scarcity of riboswitches based on the *nadA* motif could be explained by two possibilities. First, modern cells might have little need to directly monitor NAD^+^ concentrations because they use other mechanisms to adjust metabolic pathways in response to changing concentrations of this enzyme cofactor. However, this seems highly unlikely due to the great importance of NAD^+^ in many metabolic pathways spanning biosynthesis, degradation, and energy management processes (Clarke and Dafforn 1998). Second, it is possible that the task of sensing NAD^+^ is distributed over multiple different systems, including diverse protein factors, and possibly also by additional NAD^+^ riboswitch classes that remain to be discovered (Malkowski et al. 2019).

Given the importance of NAD^+^ and related compounds, and given the dearth of known riboswitches for this coenzyme, we were particularly intrigued by the discovery of a structured RNA domain that we named the “*pnuC* motif” (KI Brewer, EB Greenlee, G Higgs, et al., in prep.). This name was chosen because representatives commonly reside in the 5′ untranslated regions of open reading frames annotated as *pnuC*. These genes code for membrane proteins that presumably transport the nucleoside derivative nicotinamide riboside (NR) (Zhu et al. 1989; Kemmer et al. 2001; Sauer et al. 2004). Thus, the *pnuC* motif consensus might reflect the essential features of an aptamer domain for a novel riboswitch class that senses NAD^+^ or a related compound.

In this report, we present bioinformatic and biochemical data indicating that *pnuC* motif RNAs function as riboswitches selective for NAD^+^ and its NR-containing fragments. RNA constructs carrying the consensus *pnuC* motif undergo substantial folding changes that are induced only by their cognate ligands. Biochemical analysis of analogs indicates that the aptamer selectively recognizes functional groups on the nicotinamide-derived nucleobase. This latter finding demonstrates that natural RNAs directly recognize the redox-active moiety of this coenzyme and its immediate precursors. These findings add to the number and functional diversity of bacterial RNAs that monitor the levels of the ubiquitous coenzyme NAD^+^.

## RESULTS AND DISCUSSION

### A riboswitch candidate called the *pnuC* motif is associated with nicotinamide riboside transport

**FIGURE 1.**
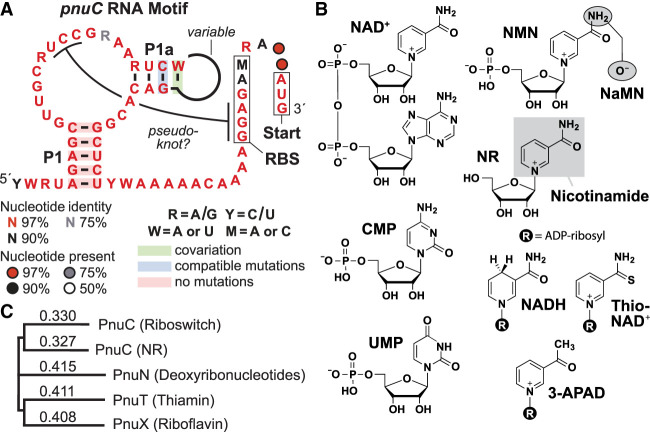
The *pnuC* motif and candidate riboswitch ligands. (*A*) Consensus sequence and secondary structure model for *pnuC* motif RNAs. The consensus was generated based on the comparison of 43 representatives with distinct sequences spanning the putative aptamer region (Supplemental Fig. S1). The boxed nucleotides near the 3′ end identify nucleotides serving as the ribosome binding site (RBS) and start codon. (*B*) Chemical structures of NAD^+^ and various natural or synthetic analogs. 3-APAD is 3-acetylpyridine adenine dinucleotide. (*C*) Comparison of the amino acid sequences of five proteins by Clustal Omega: PnuC associated with a *pnuC* motif RNA from *Streptococcus sp.* HMSC070B10, a PnuC from *Haemophilus influenzae* proven to transport NR (Herbert et al. 2003), a PnuT thiamin transporter (Jaehme et al. 2018), a PnuX riboflavin transporter from *Corynebacterium glutamicum* (Vogl et al. 2007), and a PnuN protein from *Lactobacillus acidophilus* predicted to transport a deoxynucleoside (Rodionov and Gelfand 2005). Depicted is an unrooted phylogenetic tree indicating that the riboswitch-associated PnuC protein is most homologous to a PnuC protein known to transport NR. The numbers are unitless representations of genetic distance (lengths of *horizontal* lines), and indicate the number of substitutions as a proportion of the alignment length.

A novel riboswitch candidate called the *pnuC* motif (Fig. 1A) was discovered (KI Brewer, EB Greenlee, G Higgs, et al., in prep.) by using a comparative sequence analysis approach that comprehensively reveals novel structured RNA motifs within bacterial genomes of interest. Specifically, the motif was uncovered by analyzing the sequences of noncoding regions of a given genome that are both unusually long and biased in favor of G and C nucleotides (Meyer et al. 2009; Stav et al. 2019). RNAs such as riboswitches rarely overlap protein coding regions, and usually use plentiful G and C nucleotides to form their aptamer structures even when present in bacterial species that have strongly AT-rich genomes (Klein et al. 2002; Schattner 2002; Meyer et al. 2009; Stav et al. 2019). These long, GC-rich genomic regions are examined for evidence of sequence and structural similarity to the noncoding regions of all bacterial genomes. Initially, we identified approximately 130 representatives of a conserved region commonly located upstream of a gene called *pnuC*, exclusively from species in the bacterial genus *Streptococcus*. A second gene frequently annotated as “hypothetical” is also frequently associated with *pnuC* motif representatives. The function of the protein expressed by this gene is unclear, and its presence usually coincides with a transposase gene, which further obscures its biological relevance to the RNA motif. Therefore our list of ligand candidates was inspired only by the predicted function of PnuC proteins as discussed below.

The consensus model for the *pnuC* motif (Fig. 1A) was derived by examining 43 unique-sequence representatives spanning only the most prominently conserved region (Supplemental Fig. S1). Given the small number of highly similar examples, most nucleotide positions appear to be highly conserved. Although we predict that three base-paired substructures are formed, called P1, P1a, and a possible pseudoknot (pk), only stem P1a is supported by evidence of nucleotide covariation consistent with base-pairing. This stem also appears to carry a variable loop region whose sequence is not important for the function of the RNA. Although the proposed P1 and pseudoknot substructures lack evidence of covariation, these structures are consistent with structural probing data as described in detail in a later section.

PnuC proteins encoded by *pnuC* genes associated with the newly discovered RNA motif are annotated as transporters for NR (e.g., see Zhu et al. 1989; Kemmer et al. 2001; Sauer et al. 2004). NR is a component of the ubiquitous enzyme cofactor nicotinamide adenine dinucleotide (NAD^+^) (Fig. 1B). Also, the possible pk interaction involves nucleotides predicted to serve as the ribosome binding site (RBS) for the adjacent coding region. This suggests that *pnuC* motif RNAs are likely to be genetic “OFF” riboswitches. Therefore, we speculated that the *pnuC* motif might represent the conserved aptamer domain of a novel riboswitch class that senses and responds to a molecule in the NAD^+^ biosynthetic pathway. However, various homologs of PnuC proteins have been proven to transport other metabolites such as thiamin (PnuT) (Jaehme et al. 2018) and riboflavin (PnuX) (Vogl et al. 2007), or have been proposed to transport deoxyribonucleosides (PnuN) (Rodionov and Gelfand 2005). The existence of these other proteins left open the possibility that the *pnuC* genes associated with this novel RNA motif were misannotated.

To assess this possibility, we conducted an analysis of the amino acid sequences of representative transporter proteins in this collection. Multiple sequence analysis using Clustal Omega (Sievers et al. 2011) revealed that the amino acid sequence of a randomly chosen PnuC protein associated with the newly found RNA motif (from *Streptococcus sp.* HMSC070B10) was most similar to a protein proven to selectively transport NR (Vogl et al. 2007) compared to the other transporter types (Fig. 1C). Similar results were observed by implementing an analysis using protein BLAST (Supplemental Fig. S2).

Indeed, some of the more distantly related PnuT and PnuX proteins are the products of genes occasionally found associated with riboswitches for TPP and FMN, respectively (data not shown). Thus, the specificities of the PnuC-like transporters can be predicted by the riboswitch class regulating their respective genes, and therefore it is logical that genes coding for NR transporters would be associated with a riboswitch class for NAD^+^. Therefore, we focused our biochemical analyses on assessing the ligand binding function of NAD^+^ and its derivatives with constructs carrying the *pnuC* motif consensus.

### RNA constructs carrying a *pnuC* motif bind NAD^+^ and related compounds

**FIGURE 2.**
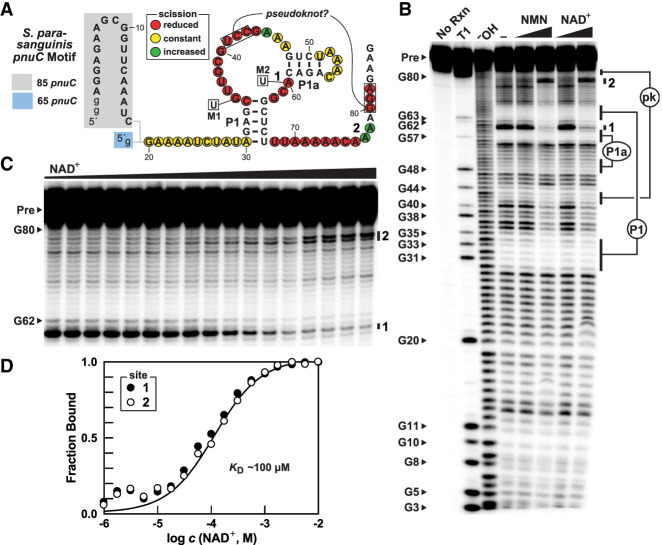
Aptamer function by *pnuC* RNA constructs. (*A*) Sequence and proposed secondary structure of RNA constructs based on the *pnuC* motif from *S. parasanguinis*. The distinct 5′ ends of the 85 *pnuC* and 65 *pnuC* constructs are highlighted, wherein the lowercase “g” letters denote guanosine nucleotides added to facilitate preparation by in vitro transcription. Colored circles identify the patterns of spontaneous RNA strand scission for nucleotides 20 through 80 resulting from in-line probing assays with the 85 *pnuC* construct as depicted in *B*. Nucleotide changes made to create mutant constructs M1 and M2 are depicted in boxes. (*B*) PAGE analysis of in-line probing reactions conducted with 5′ ^32^P-labeled 85 *pnuC* construct RNAs. No Rxn, T1 and ^−^OH identify RNAs subjected to no reaction, partial digestion with RNase T1 (cleaves after G nucleotides), or to alkali conditions (cleaves after each nucleotide), respectively. The full-length precursor RNA (Pre) and various RNase T1 cleavage products are annotated. Nucleotide regions involved in predicted base-paired structures are annotated P1, P1a, and pk (pseudoknot). Ligands were added to a final concentration of 0.1 or 1 mM. (*C*) PAGE analysis of in-line probing reactions conducted with 5′ ^32^P-labeled 65 *pnuC* construct RNAs with concentrations of NAD^+^ ranging from 1 µM to 10 mM to represent each 0.25 log M concentration units. (*D*) Plot of the fraction of RNA bound to ligand versus the logarithm of the ligand concentration (*c*). Values for fraction bound were estimated from the data in *C* based on the ligand intensities at the sites indicated. Other annotations are as described in *B*.

Initial RNA constructs used to assess ligand binding function were prepared from a representative *pnuC* motif present in *Streptococcus parasanguinis*. RNA constructs called 85 *pnuC* or 65 *pnuC* (Fig. 2A) carrying either 85 or 65 nt of the natural genomic sequence, respectively, were synthesized by in vitro transcription. These were 5′ ^32^P-labeled, and subsequently subjected to in-line probing analysis (Soukup and Breaker 1999; Regulski and Breaker 2008) to monitor changes in RNA structure brought about by ligand binding. The lengths of these constructs were chosen to evaluate the importance of nucleotides located immediately upstream of the proposed P1 stem, which was of interest due to the possible presence of conserved nucleotides.

In-line probing reactions conducted with 85 *pnuC* RNA revealed that NAD^+^ and NMN both induce substantial changes in the pattern of spontaneous RNA strand scission (Fig. 2B), which is indicative of structural reorganization brought about by ligand binding. Furthermore, no modulation is observed when the enzyme cofactors thiamin pyrophosphate (TPP) or flavin mononucleotide (FMN) are introduced (Supplemental Fig. S3). These results are consistent with the hypothesis that PnuC proteins associated with *pnuC* motif representatives are most likely NR transporters and that gene expression should be regulated by a compound indicative of the status of cellular NAD^+^ concentration.

The structural changes induced by NAD^+^ and NMN only begin with nucleotides forming the junction between P1 and P1a of the 85 *pnuC* construct (Fig. 2A), suggesting that the first ∼30 nt of the 85 *pnuC* RNA construct are not important for ligand binding. Similarly, in-line probing reactions conducted with the 65 *pnuC* RNA also exhibit robust changes to the banding pattern with NAD^+^, NMN, and NR, but not with nicotinic acid (NA) (Supplemental Fig. S4). Both constructs exhibit a pattern of bands from in-line probing reactions that are consistent with the predicted base-pairing interactions, including the pk substructure. These results suggest that the aptamer domain is confined to the most highly conserved part of the RNA. Both the nicotinamide and ribose moieties appear to carry the most important contacts for recognition by the aptamer. Moreover, the structure formed in the presence of NAD^+^ is consistent with a riboswitch mechanism wherein the ribosome binding site (RBS) is sequestered to suppress gene expression when ligand is bound (Fig. 1A).

We subjected the 65 *pnuC* RNA construct to in-line probing analysis under various concentrations of NAD^+^ to establish the apparent dissociation (*K*_D_) value for the RNA-cofactor interaction (Fig. 2C). The fraction of RNA bound to ligand was estimated by quantifying the band intensities at several key sites. By plotting fraction bound versus the logarithm of ligand concentration, a curve that is typical of a one-to-one binding interaction resulted, with a *K*_D_ value of approximately 100 µM (Fig. 2D). Similar results were observed for the 85 *pnuC* construct (Supplemental Fig. S5), suggesting again that the shorter construct carries the complete aptamer domain. There is no indication from this data that the riboswitch aptamer senses the adenosyl moiety of NAD^+^.

The concentration of NAD^+^, which is the predominant biochemical form of the nicotinamide moiety, has been measured at ∼2.6 mM in *Escherichia coli* cells (Bennett et al. 2009). Therefore, the aptamer has an affinity for NAD^+^ that is more than two orders of magnitude better than that needed if it were to function as a genetic switch at thermodynamic equilibrium. This suggests that *pnuC* motif RNAs might function as kinetically driven riboswitches (Wickiser et al. 2005a,b; Gilbert et al. 2006; Haller et al. 2011; Lemay et al. 2011; Frieda and Block 2012). Thus, *pnuC* motif RNAs appear to serve as aptamer domains for a novel NAD^+^ riboswitch class that we are naming NAD^+^-II, given that another class (hereafter called NAD^+^-I) for this coenzyme also has been published recently (Malkowski et al. 2019).

### A NAD^+^-II riboswitch aptamer requires conserved nucleotides to selectively recognize the nicotinamide riboside domain of NAD^+^

**FIGURE 3.**
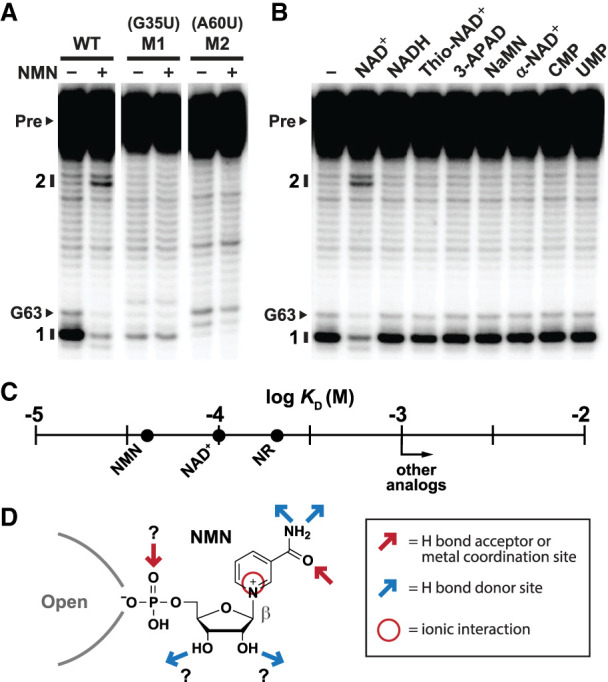
Components of the RNA and ligand essential for molecular recognition. (*A*) In-line probing assays with WT *pnuC* 65 RNA, and with mutants M1 (G35U) and M2 (A60U) in the absence (−) or presence (+) of 1 mM NMN. Additional annotations are as described for Figure 2B. (*B*) In-line probing assays with WT *pnuC* 65 RNA in the absence (−) of ligand, or in the presence of 1 mM of the compounds noted. (*C*) Plot of the *K*_D_ values determined for NAD^+^ and various analogs. Note that all compounds tested, except for NAD^+^, NMN, and NR, have *K*_D_ values poorer than 1 mM. (*D*) Model depicting possible ligand-binding interactions form NAD^+^-II riboswitch aptamers based on the existing data.

To further explore the importance of conserved nucleotides and the molecular determinants for ligand binding, we examined a series of additional RNA constructs and compounds for evidence of binding. We first determined whether single-nucleotide mutations (Fig. 2A), arbitrarily chosen from the strictly conserved positions observed (Fig. 1A), would disrupt ligand binding by the 65 *pnuC* RNA. Indeed, mutant RNA constructs M1 (G35U) and M2 (A60U) fail to respond to 1 mM NMN (Fig. 3A). In contrast, the wild-type WT 65 *pnuC* RNA construct exhibits the expected changes in banding pattern generated by in-line probing. Given the rarity of this riboswitch class, however, we cannot be certain at this time that all nucleotide positions with conserved sequence identity are essential for ligand binding function.

Next, we screened additional NAD^+^ analogs for ligand binding function. Of particular interest were those analogs that carry chemical changes to the nicotinamide moiety, which appears to be necessary for ligand binding (Supplemental Figs. S3, S4). The 65 *pnuC* RNA construct strongly discriminates against all compounds tested that carry a modification to the nicotinamide ring (Fig. 3B), including the reduced form of the natural coenzyme (NADH), or a change to the amide moiety of the pyridine ring of nicotinamide (Thio-NAD^+^, 3-APAD, and NaMN) (Fig. 1B). The RNA also rejects the pyrimidine nucleotides 5′-CMP and 5′-UMP, which is important to prevent erroneous gene regulation given that these molecules and their various phosphorylated derivatives are abundant in cells (Bennett et al. 2009). As expected, inversion of the anomeric center formed between the nicotinic acid and ribose moieties, as present in α-NAD^+^, also causes a loss of ligand binding. Given the apparent lack of contributions to binding affinity by the adenosyl moiety of NAD^+^, and given the disruptive effects of even modest changes to the nicotinamide ring of the ligand, we did not test AMP, ADP, or ATP for binding activity

These findings (Fig. 3B), along with those discussed earlier (Supplemental Figs. S3. S4) indicate that the aptamer of NAD^+^-II riboswitches selectively recognizes the nicotinamide moiety joined to a ribose via a β-N-glycosidic linkage. In-line probing assays conducted at various concentrations of ligand were used to establish *K*_D_ values for the three compounds that exhibit binding activity (e.g. see Fig. 2C). These values (Fig. 3C) indicate that the 5′-AMP moiety of NAD^+^ likely does not contribute to the molecular recognition process of NAD^+^-II riboswitch aptamers. Thus, we speculate that the RNA forms a precise binding pocket for NR recognition, and that the 5′-AMP portion of NAD^+^ will protrude outward from the aptamer core and be exposed to solvent (Fig. 3D). This is opposite of the configuration observed for the first aptamer domain of NAD^+^-I riboswitches (Malkowski et al. 2019), which selectively binds the 5′-ADP moiety and leaves the nicotinamide moiety exposed to solvent (Huang et al. 2020).

### Concluding remarks

Our findings indicate that *pnuC* motif RNAs are selective sensors of NR or its 5′-modified derivatives such as NMN and NAD^+^. Given the fact that the NAD^+^ concentration measured from *E. coli* cells is vastly greater than other natural metabolites carrying a nicotinamide mononucleotide moiety (Bennett et al. 2009), the biologically relevant ligand for regulating gene expression appears to be NAD^+^. However, because other nicotinamide-containing compounds are also recognized, cells likely use NAD^+^-II riboswitches to measure the pool of natural NR derivatives and activate expression of the *pnuC* gene if these levels become too low.

The surprising exception to this ligand collection is the reduced form of the coenzyme, NADH. Perhaps the RNA aptamer exploits the positive charge in the oxidized nicotinamide ring as a molecular recognition contact (Fig. 3D). This could also explain the requirement for the ribose moiety, whose absence would eliminate the positive charge in the pyridine ring. Given this possibility, we cannot be certain that the hydroxyl groups of the ribose moiety are exploited by the aptamer as hydrogen bond donor contacts. Identifying the precise molecular recognition with greater certainty is likely to result from biophysical analyses that establish the atomic-resolution structure of the aptamer-ligand complex, as has been achieved for an NAD^+^-I riboswitch aptamer (Huang et al. 2020).

NAD^+^-II riboswitches constitute the second class that responds to this coenzyme. The first class to be reported, based on the *nadA* motif (Weinberg et al. 2017) and now called NAD^+^-I riboswitches, uses a very unusual architecture where the adenosine monophosphate (AMP) and NMN moieties appear to be bound by separate but similar aptamer domains arranged in tandem (Malkowski et al. 2019; Huang et al. 2020). The AMP moiety is selectively recognized by the first aptamer domain, whereas the NMN moiety is exposed on the surface of the aptamer. Although it has yet to be demonstrated that the nicotinamide moiety of NAD^+^ is selectively recognized by the second domain of NAD^+^-I riboswitches, this outcome seems likely due to the similarity between the tandem aptamer domains, the similarity in the chemical structures of AMP and NMN, and the fact that the NMN moiety is exposed to solvent when the AMP moiety is docked to the first aptamer.

However, there is no notable similarity between the second aptamer domain of NAD^+^-I riboswitches and the aptamer domain of NAD^+^-II riboswitches, suggesting that these RNAs will recognize the nicotinamide moiety using distinct binding pockets. In the current report, we establish that the aptamers of NAD^+^-II riboswitches indeed make direct contacts with the NR portion of the enzyme cofactor, which is required for the RNAs to distinguish NAD^+^ from all other compounds in the cell that carry an adenosyl moiety. Indeed, it might be advantageous for NAD^+^-II riboswitch aptamers to ignore the adenosyl moiety, which would allow the RNA to avoid being triggered by the many high-concentration compounds in cells that carry this same moiety.

The discovery and validation of NAD^+^-II riboswitches aids efforts to determine the function of proteins whose expression is regulated by these riboswitches. Given that PnuC-like proteins have diversified to transport various metabolites (Jaehme and Slotboom 2015), riboswitch associations can help in the process of defining the ligands for these proteins. Our findings also support the hypothesis that modern cells might use numerous different RNA architectures to sense the enzyme cofactor NAD^+^. This would help explain why a more abundantly represented riboswitch class has not been discovered for NAD^+^, but have for some other types of enzyme cofactors such as coenzyme B_12_, TPP, SAM, FMN, and others (McCown et al. 2017). If this is true, then additional rare riboswitch classes with distinct NAD^+^-binding aptamers are likely to be discovered in the future.

## MATERIALS AND METHODS

### Chemicals

All chemicals were purchased from Sigma-Aldrich except for [γ-^32^P]-ATP, which was purchased from PerkinElmer. Enzymes and oligonucleotide sources are described elsewhere.

### Bioinformatic analysis of *pnuC* motif RNAs

The collection of 130 *pnuC* motif representatives reported elsewhere (KI Brewer, EB Greenlee, G Higgs, et al., in prep.) were reduced to 43 unique representatives that were bounded by the nucleotides beginning near the left shoulder of P1 and the start codon. Alignments were manually prepared and analyzed to establish the consensus sequence and structural model. Given the small number of unique representatives, base-pair annotations were made if any evidence of covariation was observed, without statistical analysis.

### Bioinformatic analysis of PnuC-like proteins

Clustal Omega was used to conduct comparative sequence analyses of a PnuC protein associated with the *pnuC* riboswitch candidate (*Streptococcus sp.* HMSC070B10) in comparison to transporters for NR (PnuC (Nicotinamide Riboside); *Haemophilus influenzae*), deoxynucleosides (PnuN; *Lactobacillus acidophilus*), thiamin (PnuT; *Shewanella woodyi*), and riboflavin (PnuX; *Corynebacterium glutamicum*). An unrooted phylogenetic tree was established based on the resulting sequence alignments.

Pairwise analyses of the sequences of the same proteins were also conducted by using BLASTP (McGinnis and Madden 2004). A FASTA file of the PnuC protein sequence associated with a *pnuC* motif RNA served as the query, which was compared pairwise with all other protein sequences (Supplemental Fig. S2).

### RNA oligonucleotide preparation

RNA oligonucleotides were prepared as previously described (Malkowski et al. 2019) using the appropriate synthetic DNA (Integrated DNA Technologies) templates for in vitro transcription (Supplemental Fig. S6). Template DNAs were hybridized to single-stranded T7 RNA polymerase promoter strands in transcription reactions, which were incubated at 37°C for 2–3 h. The resulting RNA transcripts were separated by using denaturing (8 M urea) 10% polyacrylamide gel electrophoresis (PAGE). RNAs were recovered from the gel, dephosphorylated using rAPid alkaline phosphatase (Roche Applied Science), and subsequently 5′ ^32^P-labeled using [γ-^32^P]-ATP and T4 polynucleotide kinase (New England Biolabs) according to the manufacturer's protocols.

### In-line probing analyses

In-line probing reactions were performed as previously described (Soukup and Breaker 1999; Regulski and Breaker 2008). ^32^P-labeled RNAs (trace) were incubated in the absence or presence of ligand candidates as indicated in the presence of 20 mM MgCl_2_, 100 mM KCl, and 50 mM Tris-HCl (pH 8.3 at ∼23°C). The reaction products were separated by denaturing (8 M urea) 10% PAGE and were visualized by using a Typhoon phosphorimager (GE Healthcare). As described previously (Malkowski et al. 2019), band intensities were determined and used to estimate the fraction of RNAs bound to ligand. Values were plotted relative to the logarithm of the molar concentration of ligand, wherein half-maximal binding represents the *K*_D_.

## SUPPLEMENTAL MATERIAL

Supplemental material is available for this article.

## Supplementary Material

Supplemental Material
