# Supplementary material for: A second riboswitch class for the enzyme cofactor NAD+
Source: RNA. 2021 Jan;27(1):99–105. doi: 10.1261/rna.077891.120 (PMC7749635; doi:10.1261/rna.077891.120)
Supplement: Supplemental Material [file supp_27_1_99__index.html]

A Second Riboswitch Class for the Enzyme Cofactor NAD+ — A second riboswitch class for the enzyme cofactor NAD+ — Supplemental Material 

# A second riboswitch class for the enzyme cofactor NAD+

## Supplemental Material

- Supplemental\_Figures.docx
