# Supplementary material for: A second riboswitch class for the enzyme cofactor NAD+
Source: RNA. 2021 Jan;27(1):99–105. doi: 10.1261/rna.077891.120 (PMC7749635; doi:10.1261/rna.077891.120)
Supplement: Supplemental Material [file supp_077891.120_Supplemental_Figures.docx]

**Supplemental Information**

**A Second Riboswitch Class for the Enzyme Cofactor NAD^+^**

SHANKER S. S. PANCHAPAKESAN,^1,*^ LUKAS COREY,^1,*^ SARAH N. MALKOWSKI,^2^ GADARETH HIGGS,^1^ AND RONALD R. BREAKER^1,3,4^

^1^Department of Molecular, Cellular and Developmental Biology, Yale University, New Haven, Connecticut 06520-8103, USA

^2^Department of Chemistry, Yale University, New Haven, Connecticut 06520-8103, USA

^3^Department of Molecular Biophysics and Biochemistry, Yale University, New Haven, Connecticut 06520-8103, USA

^4^Howard Hughes Medical Institute, Yale University, New Haven, CT 06520-8103, USA

**Corresponding author:** [**ronald.breaker@yale.edu**](mailto:ronald.breaker@yale.edu)

*These authors contributed equally to this work

**
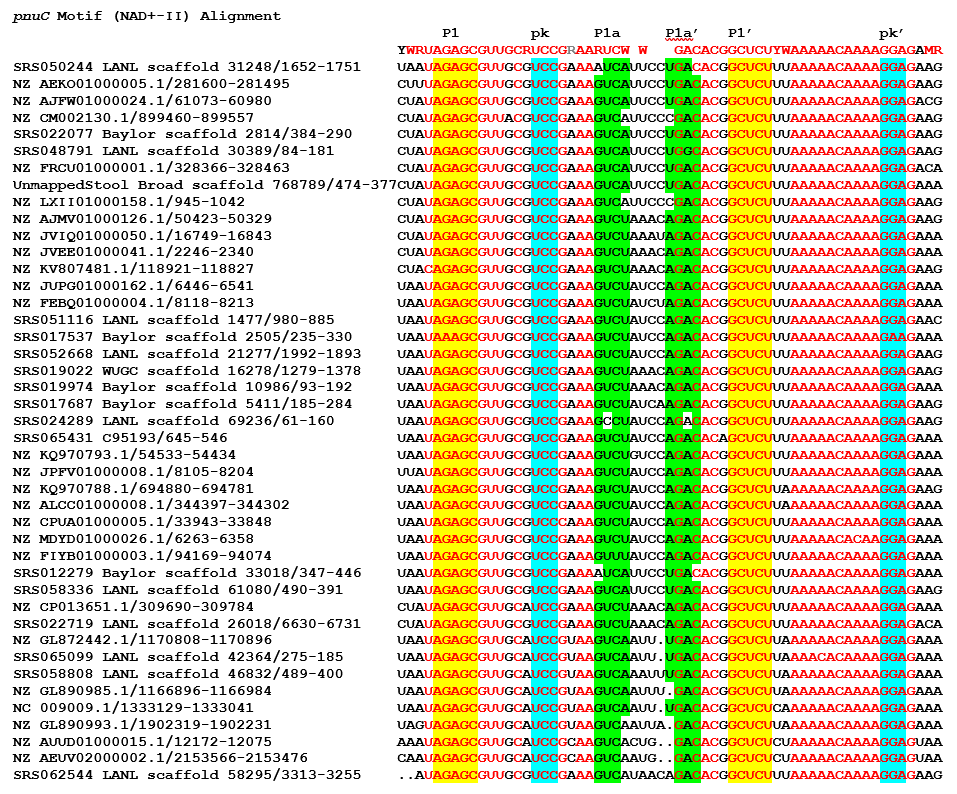
**

**FIGURE S1.** Sequence alignment of 43 *pnuC* motif representatives that have distinct sequences within the putative aptamer region depicted. Top line is the consensus sequence, wherein red letters identify nucleotide positions that have no more than one mutation relative to the nucleotide identity depicted. Color shading identifies predicted base-paired regions as annotated. Other annotations are as described in the legend to **Fig. 1**.

**A**

>PnuC-Riboswitch

MHTYLQKKIKNIKTTLGEMSGGYRRMVTAMSDLGFSGTMKAIWDDLFAHRSFAQWLYLLVLGSFPLWLELIYEHRIVDWIGMICSLTGIICVIFVSEGRASNYLFGLINSVIYLILALQKGFYGEVLTTLYFTVMQPIGLLVWIYQAQFKKEKQEFVARKLDGKGWTKYISISVLWWLAFGFIYQSIGANRPYRDSITDATNGVGQILMTAVYREQWIFWVATNVFSIYLWWGESLQIQGKYLIYLINSLVGWYQWSKVAK>

>PnuC-NR

MTLAARLKQEFVSGWKPFEVVWLALFIIAQIWAYVQTPDSWLAMISGISGILCVVLVSKGKISNYFFGLIFAYTYFYVAWGSNFLGEMNTVLYVYLPSQFIGYFMWKANMQNSDGGESVIAKALTVKGWMTLIVVTTVGTLLFVQALQAAGGSSTGLDGLTTIITVAAQILMILRYREQWLLWIGLNILSIFLWAETPAIYLMYSAYLLNSLYGYYNWTKLVKRTN>

>PnuX

MNPITELLDATLWIGGVPILWREIIGNVFGLFSAWAGMRRIVWAWPIGIIGNALLFTVFMGGLFHTPQNLDLYGQAGRQIMFIIVSGYGWYQWSAAKRRALTPENAVAVVPRWASTKERAGIVIAAVVGTLSFAWIFQALGSWGPWADAWIFVGSILATYGMARGWTEFWLIWIAVDIVGVPLLLTAGYYPSAVLYLVYGAFVSWGFVVWLRVQKADKARALEAQESVTV>

>PnuT

MTDFWSALLNTFTGALSEANALTAWEGVAVILAAAYLLLAMKGSIWCWFAAFASTAIYTALFWKVSLLMESVLNVYYMAMAIYGFQQWSKGKRDDTGGVISWSFNRHLKIIVVTAGVSLLMGYLMANFTSASFPYLDAATTCYAVMTTYLVAKKVLENWLYWVVIDLVSIYLYLQKGLMLTSLLFILYVGMAIGGYFLWRSTMREQDMATVS>

>PnuN

MERHENYFVWLFNQLKGWPVQNYCLWFFAFGFQLALLIQAKVTSVTLITFIGTLLGTLCVLAINATKAINGWLGLVSAACFIYAGWSAKNYLSIFEQIAYIATLDLPVIISVRSWNDDTKNHLRKFGAKEWVIAIVGTFLVYLVSGYLIGKFTNDPRPWVDAISFAISLTAGIMCFMRYNNQYFWWTASGIFQLILWGITYAQGDANLAMAINSLIYVINDVLAFTVSPWFNMGRRRAGLKEISK>

**B**


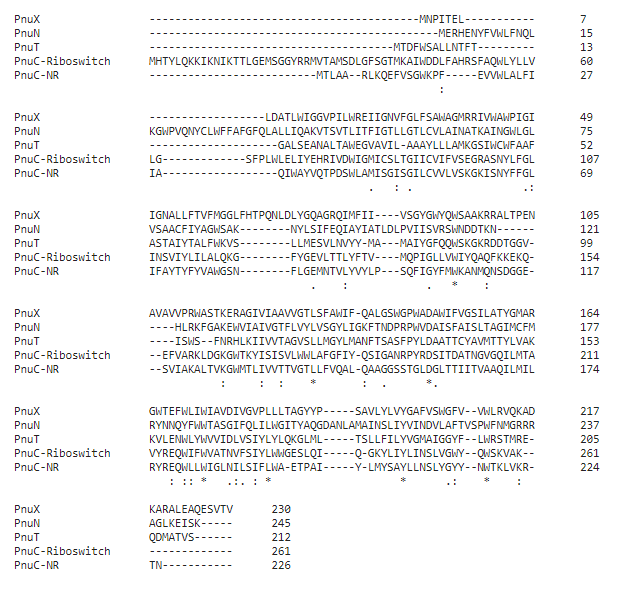


**C**


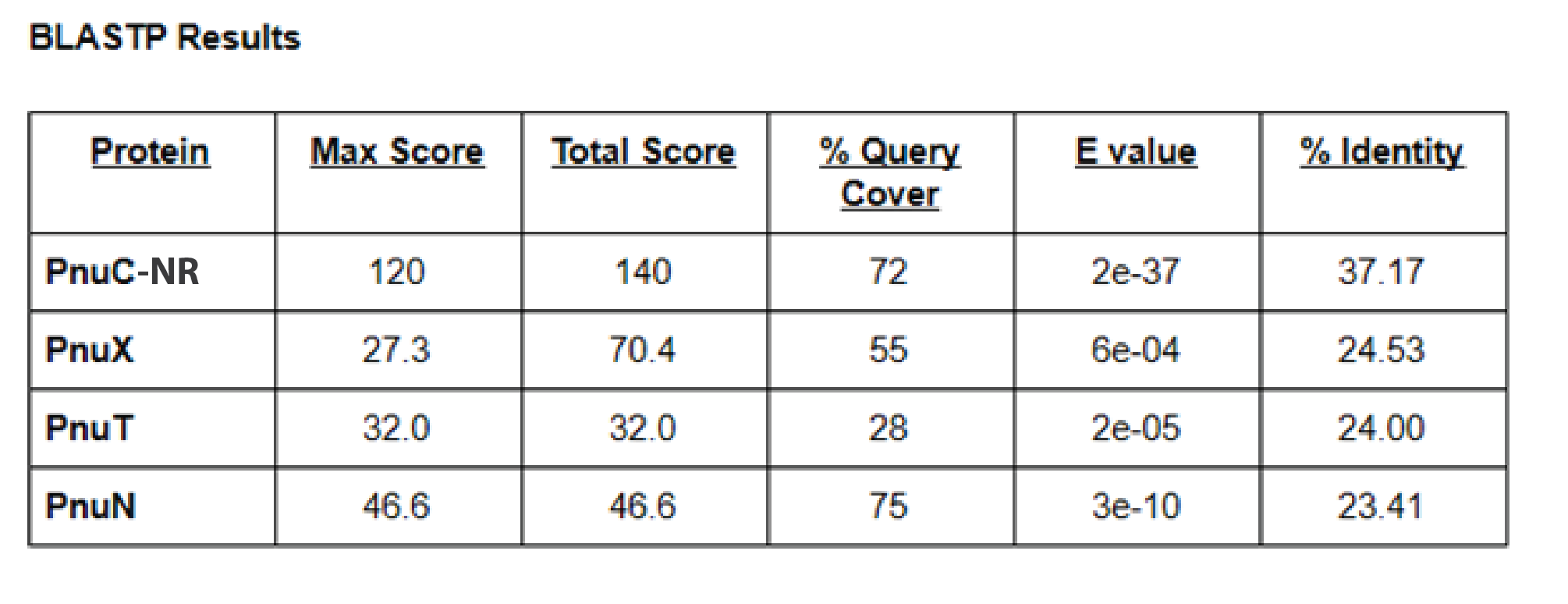


**FIGURE S2.** Clustal Omega and BLASTP analysis of PnuC-like proteins. (A) Amino acid sequences of a PnuC protein associated with a *pnuC* riboswitch (PnuC-Riboswitch) and the PnuC-like proteins known to transport NR (PnuC-NR), riboflavin (PnuX), thiamin (PnuT), and a putative transporter of deoxyribonucleotides (PnuN). (B) Clustal Omega sequence alignment of the proteins described in A. Asterisks identify amino acid positions conserved in all proteins analyzed. (C) BLASTP analysis of the proteins depicted in A. The riboswitch-associated PnuC is not depicted, and other protein names are as defined in the main text.


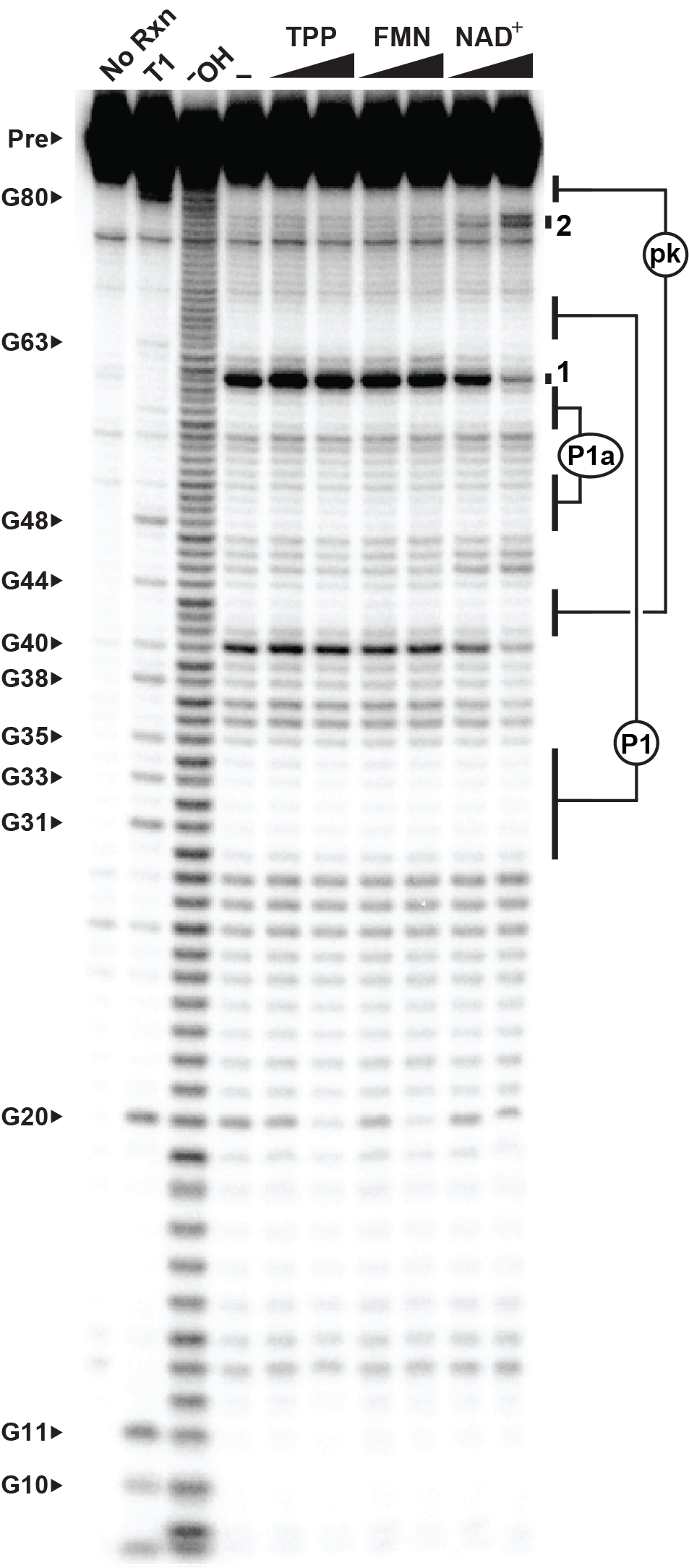


**FIGURE S3.** In-line probing assay using the 85 *pnuC* construct with ligand concentrations of 0.1 and 1 mM. Additional methods and annotations are as described for Fig. 2B.


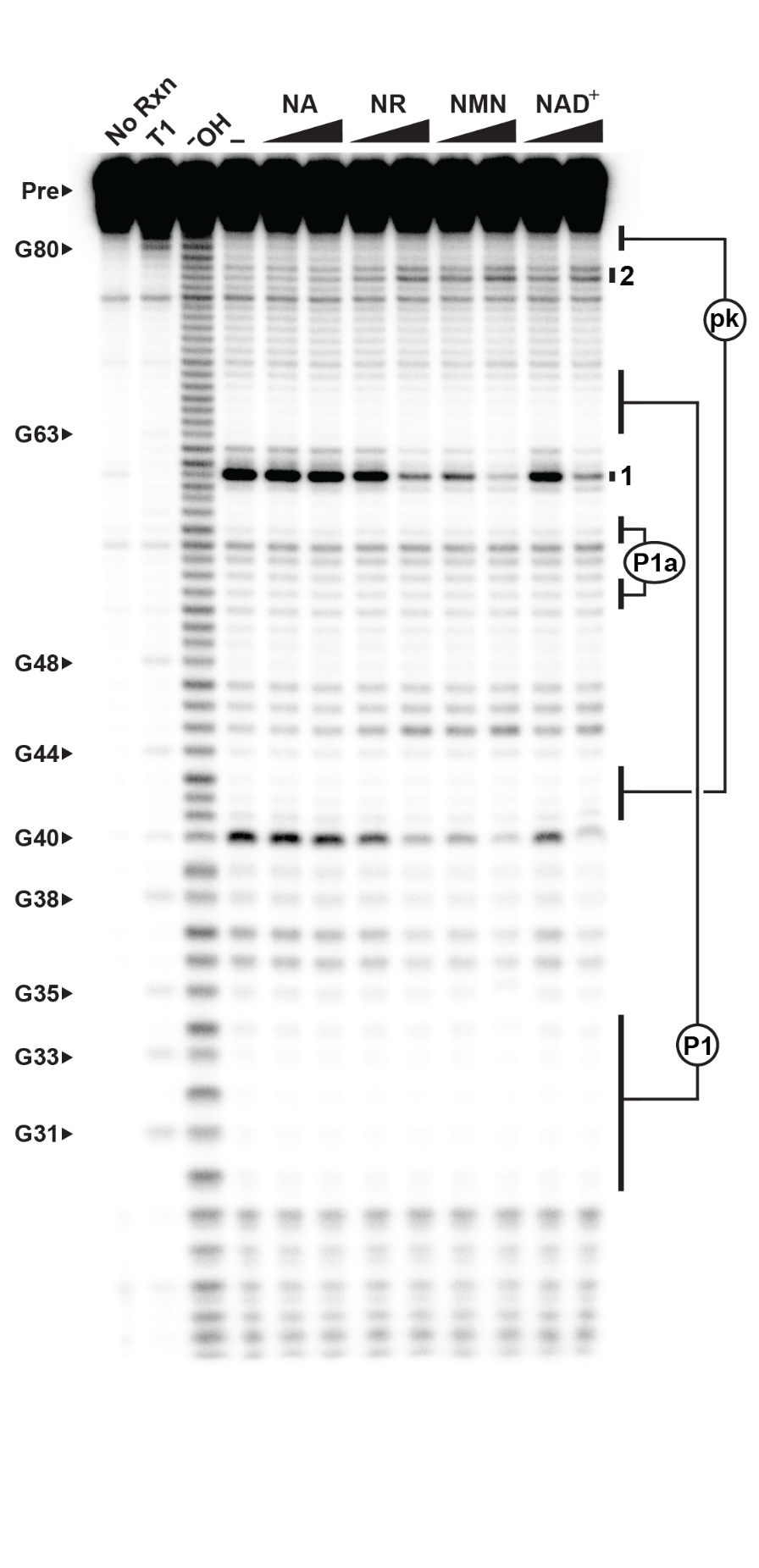


**FIGURE S4.** In-line probing assay using the 65 *pnuC* construct with ligand concentrations of 0.1 and 1 mM. Additional methods and annotations are as described for Fig. 2B.


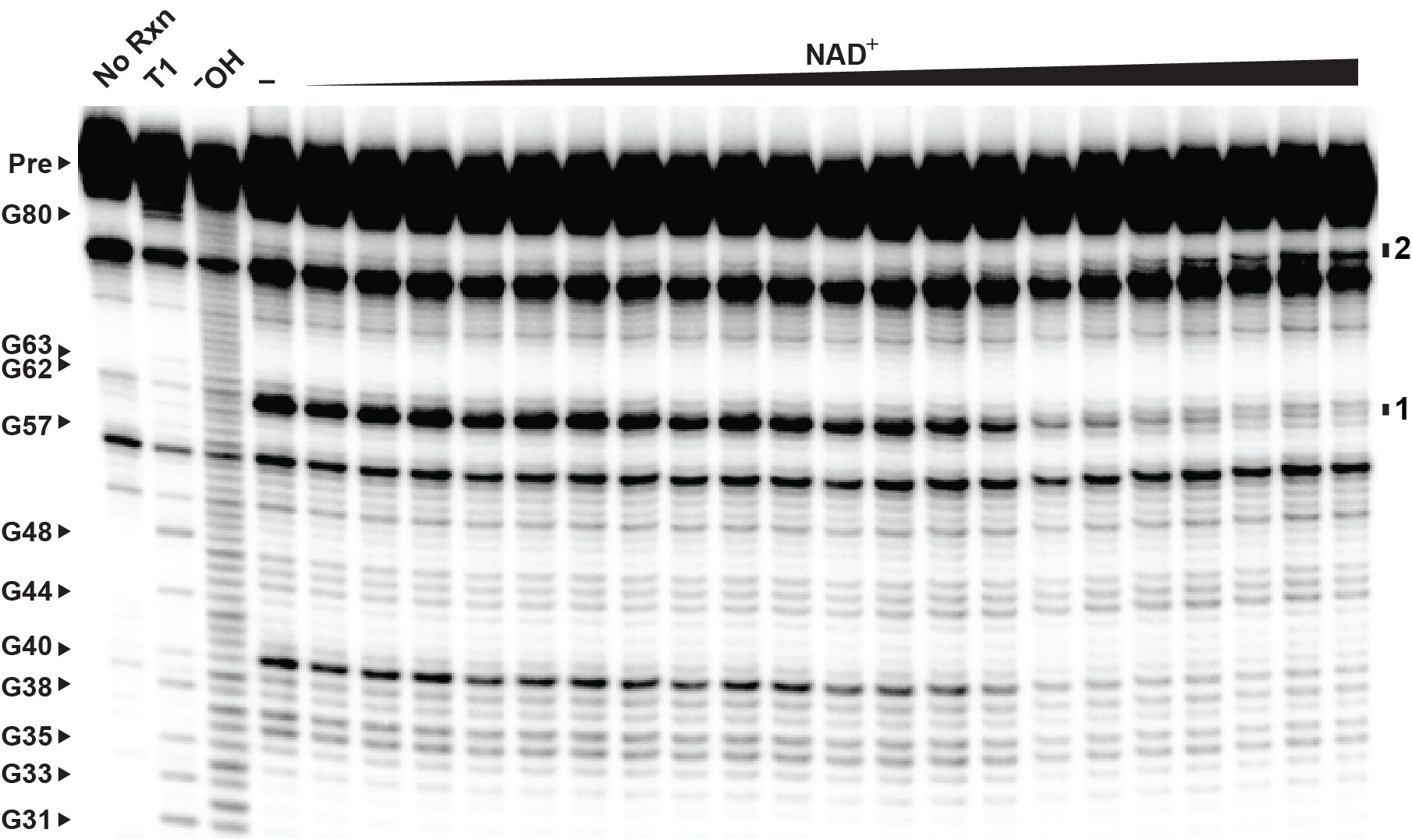


**FIGURE S5.** In-line probing assay using the 85 *pnuC* construct with concentrations of NAD^+^ ranging from 1 μM to 10 mM to represent each 0.25 log M concentration units. Annotations are as described in the legend to **Fig. 2B**.


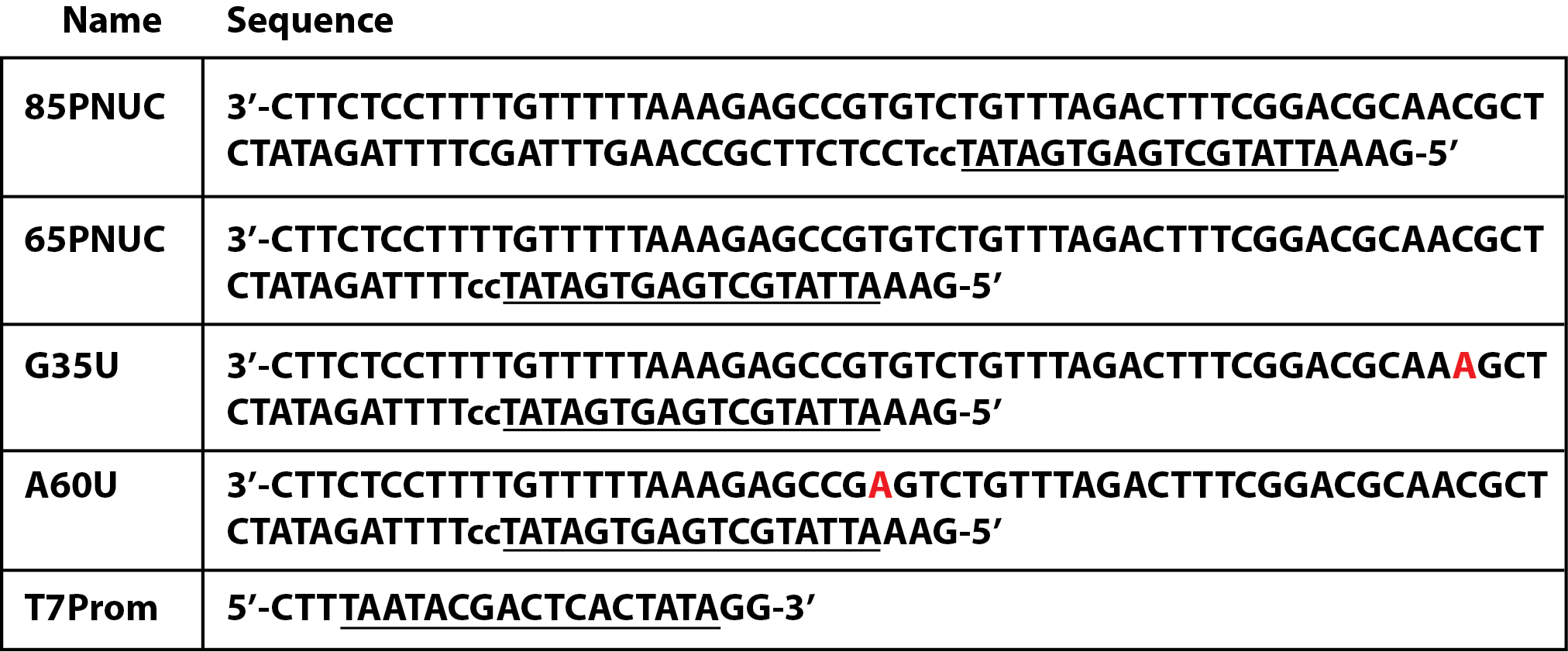


**FIGURE S6.** DNA oligonucleotides used in the current study. Template strands are depicted 3́ to 5́, whereas the sequence carrying the non-template strand of the T7 RNA polymerase promoter sequence is depicted from 5́ to 3́. Underlined nucleotides represent the 17-nucleotide promoter sequence. Red nucleotides identify the mutated template for preparation of RNA constructs M1 (G35U) and M2 (A60U).
